# Access to antiepileptic drug therapy in children in Camagüey Province, Cuba

**DOI:** 10.1111/j.2042-7174.2012.00215.x

**Published:** 2012-12

**Authors:** Zeina Bárzaga Arencibia, Alberto López Leyva, Yordanka Mejías Peña, Alba Rosa González Reyes, Maurilys Acosta Nápolez, Demetrio Carbonell Perdomo, Edita Fernández Manzano, Imti Choonara

**Affiliations:** a Department of PharmacoepidemiologyCamagüey; b Department of Paediatric Neurology, Camagüey Children's HospitalCamagüey; c Carlos J. Finlay PolyclinicCamagüey; d Provincial Pharmacovigilance CentreCamagüey; e Faculty of Pharmacy, University of HavanaHavana, Cuba; f Academic Division of Child Health, University of Nottingham, Derbyshire Children's HospitalDerby, UK

**Keywords:** access, antiepileptic drug, epilepsy

## Abstract

**Objective:**

To describe access to antiepileptic drug therapy and estimate the prevalence of epilepsy in children in Camagüey Province, Cuba.

**Methods:**

All the community pharmacies in the province were visited and information collected about the number of children receiving antiepileptic drugs in 2009. Availability and cost of each antiepileptic drug were determined. The prevalence of epilepsy was estimated by determining the number of children receiving antiepileptic drugs.

**Results:**

There were 923 children who received a total of 977 antiepileptic drugs in Camagüey Province. The estimated prevalence of epilepsy was 5.18 per thousand children which is lower than previously reported rates in other low and lower-middle income countries. Most of the children (871, 94%) received a single antiepileptic drug. Carbamazepine and valproate were the two most frequently prescribed antiepileptic drugs. Antiepileptic drugs were available from the local pharmacy on 76% of occasions. If the antiepileptic drug was not available from the local pharmacy, the parent had to travel to another pharmacy to obtain the medicine.

**Conclusions:**

The estimated prevalence of epilepsy in children in Cuba is lower than that estimated in other lower-middle income countries. Access to drug therapy in children with epilepsy can be achieved in lower-middle income countries.

## Introduction

Epilepsy is one of the most common neurological conditions affecting children worldwide. The prevalence of epilepsy in children ranges from 1.5–8 per thousand children. Most of these studies have been conducted in high-income countries.[1,2] The prevalence of epilepsy in Latin America and the Caribbean has been less extensively studied, but appears to be higher (8.7–12.4 per thousand).[3,4] The prevalence of epilepsy in children in Cuba at present is unknown.

It has been estimated that worldwide 80–85% of individuals with epilepsy fail to receive treatment.[5–8] There are many possible reasons for this. The individual may not have seen a health professional to diagnose the epilepsy or the antiepileptic drug (AED) is too expensive or not readily available. Several AEDs (carbamazepine, diazepam, lorazepam, phenobarbital, phenytoin and sodium valproate) are on the World Health Organization's List of Essential Medicines for Children.[9] The presence/absence of essential medicines for children in pharmacies has been used as a marker of the availability of essential medicines.[10]

Cuba, despite being a poor country, has an excellent healthcare system.[11] Its under-5 child mortality rate is comparable to that of high-income countries. Its success is thought to be due to two main factors: (1) an excellent primary healthcare system and (2) universal access to free health care.[11]

A National Medicines Programme involving family doctors and pharmacists was established in Cuba in 1991 to ensure the availability of essential medicines for all patients.[12] Since 1994, patients with epilepsy are registered.[12] Following the diagnosis of epilepsy by the provincial paediatric neurology team at the Provincial Children's Hospital in Camagüey, Cuba, parents receive a medical certificate. This certificate states the diagnosis, the AED that the patient is to receive, the dose and duration of treatment (maximum of 1 year, as all children with epilepsy are reviewed at least once a year by the provincial paediatric neurology team). Subsequently, the parents can present their certificate at a community pharmacy in order to purchase the AED for their child without the need for a further prescription.

There are three types of community pharmacies in Cuba. Each municipality (and each of the four districts in Camagüey Municipality) has a main pharmacy. The next level of pharmacy is the special pharmacy which is usually located next to a polyclinic. The number of special pharmacies in each municipality/district ranges from zero to five. The third level of pharmacy is a local pharmacy. These are usually located in isolated or rural communities and have the smallest stock of drugs. The number of local pharmacies within each municipality/district ranges from one to 14. The cost of any medicine is identical at all community pharmacies throughout the country. Like other medicines in Cuba, AEDs need to be purchased at a minimal cost. Topiramate and two formulations of sodium valproate (tablet or a suspension of 200 mg/5 ml) are not available at community pharmacies. They are only available from the hospital pharmacy and because parents have to travel to the hospital pharmacy to obtain these medicines, they are dispensed free of charge.

Access to drug therapy is a key component of health care and we therefore decided to look at access to AED therapy for children with epilepsy in Camagüey Province. The primary supplier for AEDs is the community pharmacy.

## Methods

### Setting

An observational study was conducted between January and December 2009 in the Province of Camagüey. Camagüey is the largest province of the island in Cuba and is located in the central region. It is divided into 13 municipalities and the main municipality of Camagüey is subdivided into four districts. Each municipality has urban and rural areas and in 2009 the province had a population of 178 231 children under the age of 18 years. Camagüey Province has 149 community pharmacies. These include 16 main pharmacies, 31 special pharmacies and 102 local pharmacies. The area covered by each individual pharmacy can vary considerably. In urban areas pharmacies cover an average area of 20 km^2^. In rural areas they cover a larger area (up to a maximum of 200 km^2^). Distances between pharmacies range from 20 m in an urban setting near a hospital (the minimum) and 50 km in rural areas.

### Data collection

Each individual community pharmacy has a Pharmacy Technical Director. This individual obtained data regarding the availability of each AED throughout the study period. Further information regarding stock and availability of each AED was obtained by telephone at the end of February, June and December 2009. Additionally, each of the 149 community pharmacies in the province was visited by one of the researchers on one occasion during the first 6 months of the year.

### Data

Medical certificates for patients receiving AEDs were reviewed at each individual community pharmacy. Certificates for patients under 18 years with a diagnosis of any type of epilepsy were retrieved and data regarding age, type of AEDs received and doses were collected and entered onto a secure electronic database with restricted access.

Information about patients attending pharmacies was crosschecked with the patient data stored in paper-based records at the paediatric neurology clinic at Camagüey Children's Hospital. Monthly data were collected and crosschecked from the hospital by the research team. The price for AEDs was taken from the ‘National Official Prices List to the Population’ produced by the Cuban government, and the monthly cost of treatment for children was calculated.

## Results

There were 923 children with epilepsy receiving a total of 977 AEDs identified. This is a prevalence of 5.18 per thousand children. Eight hundred and seventy-one children were on monotherapy, 50 on two AEDs and two on three AEDs. The age distribution of the children with epilepsy and the prevalence in relation to age is shown in Table 1. Crosschecking identified five children who had attended the paediatric neurology clinic but had not registered with a community pharmacy. These children were initially receiving AEDs from another family member but after discussion agreed to register at a community pharmacy. The number of children with epilepsy and the number of pharmacies in each municipality/district is shown in Table 2. The number of children with epilepsy per pharmacy ranged from 0–37.

**Table 1:** Age and prevalence of children with epilepsy in Camagüey Province, Cuba

Age of children	Number of children with epilepsy	Number of children in province	Prevalence of epilepsy per 1000 children
0–11 months	4	8 387	0.48
1–2 years	61	14 887	4.10
3–5 years	111	25 589	4.34
6–10 years	345	47 951	7.19
11–18 years	402	81 417	4.94
**Total**	**923**	**178 231**	**5.18**

**Table 2:** Demographic information of children with epilepsy and community pharmacies in Camagüey Province, Cuba

Municipalities	Child population	Number of children with epilepsy	Total number of pharmacies
Camagüey			
A	18 838	101	7
B	17 261	64	5
C	9 853	92	11
D	24 720	132	12
Florida	15 763	61	17
Esmeralda	7 146	35	8
Vertientes	12 978	62	10
Céspedes	5 956	47	5
Minas	8 926	47	6
Santa Cruz del Sur	12 310	18	13
Jimaguayú	5 548	54	8
Sibanicú	7 552	53	7
Sierra de Cubitas	4 748	20	8
Nuevitas	3 656	71	8
Guáimaro	12 979	53	15
Najasa	3 997	13	9
**Total**	**178 231**	**923**	**149**

Carbamazepine was the most widely prescribed AED but was not available as a suspension (Table 3). Sodium valproate was the second most frequently prescribed AED but was only available from community pharmacies as a syrup in a concentration of 125 mg/5 ml. The majority of children who required valproate as a tablet received magnesium valproate.

**Table 3:** Cost and availability of antiepileptic drugs in community pharmacies in Camagüey Province, Cuba

Drug	Pharmaceutical formulation	Price for a month's supply (Cuban pesos)	Quantities of weeks in which the AED was out of stock	Number of children
Carbamazepine E	90 tablets 200 mg E	4.50	0	480
Clonazepam	50 tablets 1 mg	5.30	0	31
Ethosuximide	30 capsules 250 mg†	13.80	0	4
Lamotrigine	30 tablets 100 mg†	12.00	0	10
Phenytoin E	50 chew tablets 50 mg E	1.60	0	0
Phenytoin	230 ml suspension 125 mg/5 ml	6.00	0	37
Phenobarbitone E	60 ml elixir 15 mg/ml E	2.10	0	0
Phenobarbitone E	10 tablets 100 mg E	0.45	4	46
Phenobarbitone E	20 tablets 15 mg E	0.60	0	0
Primidone	10 tablets 0.25 mg	0.90	0	0
Primidone	115 ml suspension 200 mg/ml	1.80	0	0
Topiramate	10 tablets 100 mg†	Free‡	0	7
Magnesium valproate	30 tablets 190 mg	1.00	5	163
Sodium valproate	120 ml syrup 125 mg/5 ml	10.00	2	156
Sodium valproate	120 ml suspension 250 mg/5 ml	Free‡	0	10
Sodium valproate E	100 tablets 500 mg E	Free‡	0	15
Vigabatrin	60 tablets 500 mg	18.00	0	18

Exchange rate of pesos to euros (12/2009): 1 Cuban peso = 0.0551 euro.

† = Imported; ‡ = from hospital pharmacy only; AED, antiepileptic drug; E = essential medicine (on World Health Organization List of Essential Medicines for Children^9^).

Most of the AEDs were available from all levels of pharmacy. The price of a month's supply of AEDs ranged from being freely available (sodium valproate suspension and tablets and topiramate tablets) to 18 Cuban pesos (CUPs) (vigabatrin) (Table 3). On 24% of occasions an AED was not available at the local pharmacy. Three formulations of AEDs were not available within the community pharmacies for between 2 and 5 weeks (Table 3). They were, however, available in the hospital pharmacies.

## Discussion

The estimated prevalence of epilepsy in children in Camagüey Province (5.18 per thousand children) is lower than the prevalence in children and adults throughout Latin America (12.4)[3] and in Brazilian children living in an area of high deprivation (8.7).[4] It is, however, similar to the only previous study (published 30 years ago) looking at the prevalence of children with epilepsy in Cuba (7.5).[13]

A limitation of this study is that the prevalence of epilepsy was estimated by the number of children receiving AEDs. Others have used questionnaires looking at the entire population.[4] Using the number of patients receiving AEDs has previously been used in countries with good primary health care (Sweden and Denmark).[1,2] Cuba has an excellent primary health care system.[11] All children have a family doctor who routinely sees every child twice a year.[11] This excellent system of primary health care, alongside integration between primary and secondary health care ensures that children with epilepsy are identified.

The low prevalence of epilepsy in Cuba has previously been noted.[3] In children, contributory factors include: a comprehensive immunisation schedule; excellent antenatal care that is associated with a low prevalence of low birth weight babies;[14] a low incidence of infectious diseases such as malaria, tuberculosis, schistosomiasis and cysticercosis.[15]

The vast majority of the children (94%) were on a single AED only, which is widely accepted as best clinical practice. Carbamazepine and valproate were the two most frequently prescribed AEDs and they are the medicines recommended as first-line therapy, in both Cuba and the UK.[16] Many children received magnesium valproate which is not extensively used in Europe. It is, however, widely used in Latin America and has been shown to have bioequivalence and similar efficacy to sodium valproate.[17–19] It is considerably cheaper than sodium valproate and may actually have some advantages in that there is less inter-individual variation in plasma concentrations of valproic acid with magnesium valproate.[17]

Access to medicines is a significant problem in low and lower-middle income countries. A study of 14 countries in Central Africa identified a significant lack of essential medicines for children in pharmacies and central medical stores with availability in retail or private pharmacies ranging from 38–62%.[10] A recent study of epilepsy in Africa found that 30% of the patients with epilepsy and 73% of children never received treatment.[20]

Access to AEDs in pharmacies in Camagüey is generally good for a lower-middle income country in that on 76% of occasions the AED was available. The USA has imposed an economic blockade on Cuba for the last 50 years, which makes it more difficult to obtain medicines as well as many items involved in the transportation of goods.[21]

Three different formulations of valproate and phenobarbitone (sodium valproate syrup, magnesium valproate tablets and phenobarbitone tablets) were not available for between 2 and 5 weeks in the community pharmacies. All three formulations were, however, available at the hospital pharmacy. Hospital pharmacies will dispense AEDs free of charge in an emergency situation, i.e. if the community pharmacy does not have the AED in stock. The hospitals have enough medicines within their storerooms for at least two months. The most significant shortage related to magnesium valproate tablets, which were not available for 5 weeks.

Parents could go to an alternative pharmacy (including the hospital pharmacy) if the AED was unavailable or wait for a maximum of up to 2 days for the pharmacy to arrange transfer of the appropriate formulation from another pharmacy within the province. There is a weekly meeting within the province between the head of pharmacy and the companies involved in the distribution and storage of medicines to try and ensure that children continue to receive treatment.

Despite the successes of ensuring that children receive AED therapy, there are clearly issues that could be improved. Carbamazepine is not available in a liquid formulation. The only medicines available in a liquid formulation are sodium valproate, which is not recommended for children under the age of 3 years because of the risk of hepatotoxicity and the older AEDs (phenytoin, phenobarbitone and primidone) all of which have a higher incidence of side effects. Although health care itself is free within Cuba, parents still have to pay for AEDs for their children with epilepsy. The problems in Cuba in relation to access to AED therapy, however, are minor in comparison to many other lower middle-income countries throughout the world. The stigma of epilepsy is a major problem in many countries.[7] Universal education plays a major role in combating stigma. As well as combating stigma, health professionals and health systems need to ensure that AEDs are readily available at pharmacies that are accessible and that the AEDs are sold at a price that is affordable to the majority of the population.

## Conclusions

The prevalence of epilepsy in children in Cuba is lower than that estimated in other lower middle-income countries. It is possible for lower middle-income countries to ensure that children with epilepsy receive regular AED therapy.
